# Ultrasound-guided management in heart failure: A systematic review and meta-analysis evaluating lung and inferior vena cava ultrasound for reducing readmissions and mortality

**DOI:** 10.1016/j.ahjo.2026.100830

**Published:** 2026-07-06

**Authors:** Amir Behzad Bagheri, Muhammad Saleem, Sina Bakhshaei, Raymond Andrew Dieter, Ali Ajam, Yasaman Navari, Benay Ozbay, Soham Nadkarni, Parth Patel, Mobin Kheirkhahan, John Jude Pacella, Babak Razani

**Affiliations:** a Department of Medicine, University of Tennessee Graduate School of Medicine, Knoxville, TN, USA; b Department of Medicine, University of Pittsburgh Medical Center, Pittsburgh, PA, USA; c Hurley Medical Center/Michigan State University, Flint, MI, USA; d Department of Cardiology, UHS Southwest Healthcare MEC, Temecula, CA, USA; e Division of Cardiology, University of Tennessee health Science Center, Knoxville, TN, USA; f Department of Medicine, Vascular Medicine Institute, University of Pittsburgh School of Medicine and UPMC, Pittsburgh, PA, USA; g Pittsburgh VA Medical Center, Pittsburgh, PA, USA; h Department of Medicine, Heart and Vascular Medicine Institute, University of Pittsburgh School of Medicine and UPMC, Pittsburgh, PA, USA; i University Heart, University of Mississippi Medical Center, Jackson, MS, USA

**Keywords:** Heart failure, Heart failure exacerbation, Point-of-care ultrasound, POCUS, Randomized controlled trials

## Abstract

**Importance:**

Hospital admissions due to HF exacerbations often occur due to vascular congestion. With point-of-care ultrasound (POCUS) becoming more prevalent, this systematic review was performed to identify its utility by using lung and/or IVC ultrasound to visualize vascular congestion to decrease all-cause mortality and/or hospital readmissions.

**Objective:**

To determine whether ultrasound-guided management using lung and/or IVC ultrasound reduces hospital readmissions and all-cause mortality in patients with HF compared to standard care.

**Data sources:**

Seven RCTs, published between 2019 and 2024, were identified through systematic searches of PubMed, Embase, Web of Science, and Google Scholar.

**Study selection:**

Two reviewers independently screened studies using PRISMA 2020 guidelines. Eligible studies were RCTs enrolling adults with HF in which diuretic therapy was guided by lung and/or IVC ultrasound, compared to standard care, reporting all-cause mortality or HF-related readmission. Studies were excluded if non-randomized, non-English, echocardiography-only, or involving pediatric populations.

**Data extraction and synthesis:**

Data extraction was manually performed and included study characteristics, participant demographics, ultrasound modality, and outcome measures. A DerSimonian–Laird random-effects model was applied to account for clinical and methodological heterogeneity.

**Main outcome(s) and measure(s):**

The primary outcomes, planned prior to data collection, included all-cause mortality and hospital readmissions for patients admitted for HF exacerbations when utilizing ultrasound-guided management versus standard care. The data was pooled and measured using a random-effects model to calculate RR with 95% confidence intervals (CI).

**Results:**

From the seven RCTs (*n* = 824), ultrasound-guided management significantly reduced HF readmissions (RR = 0.61; 95% CI 0.38–0.96; *p* = 0.034; I^2^ = 52%), corresponding to a 39% risk reduction, with a numerically larger reduction observed in the exploratory subgroup of combined lung + IVC ultrasound (RR = 0.28; 95% CI 0.13–0.60; *p* = 0.001; 2 trials, *n* = 65) compared to lung ultrasound alone (RR = 0.75; 95% CI 0.48–1.17; *p* = 0.211; 5 trials), though the subgroup interaction test was not statistically significant and this finding should be considered hypothesis-generating. No significant reduction in all-cause mortality was observed (RR = 0.96; 95% CI 0.58–1.58; *p* = 0.859; I^2^ = 9%).

**Conclusions and relevance:**

Seven RCTs totaling 824 patients showed that ultrasound-guided HF management significantly cut readmission risk without improving survival. A larger numerical reduction in the combined LUS + IVC subgroup is exploratory only based on two small trials and requires prospective confirmation before influencing clinical practice.

## Highlights

• **Question**: Does ultrasound-guided management reduce readmissions and all-cause mortality in heart failure versus standard care?
• **Findings**: Ultrasound-guided management significantly reduced HF readmissions (39% risk reduction) but not all-cause mortality.
• **Meaning**: Ultrasound-guided decongestion reduces HF rehospitalizations; combined lung plus IVC showed greater benefit than lung alone.

## 1 Introduction

Vascular congestion is the leading cause of hospitalizations in patients with heart failure (HF) and is strongly associated with recurrent readmissions and adverse outcomes. Despite advances in therapy, nearly one in four HF patients is readmitted within 30 days of discharge, highlighting the ongoing burden of recurrent hospitalization [1], [2]. Reliance on clinical signs alone is limited, as jugular venous distension and pulmonary rales correlate poorly with invasive hemodynamics [3]. Current guidelines emphasize the need for objective bedside tools to optimize decongestion and reduce rehospitalizations [4].

Ultrasound-guided (USG) management offers real-time, non-invasive assessment of congestion. Lung ultrasound (LUS) detects interstitial fluid via B-lines and has shown superior sensitivity to chest radiography in identifying pulmonary congestion [5], [6], [7]. In the randomized LUS-HF trial [8], LUS-guided therapy in ambulatory HF patients significantly reduced urgent visits and hospitalizations when compared to standard care. In contrast, the BLUSHED-AHF trial [9], which was conducted in the emergency department, did not demonstrate improvement in early outcomes despite greater reductions in B-lines. These discrepant findings highlight the influence of patient population, timing, and protocol heterogeneity on trial results.

Inferior vena cava (IVC) ultrasound provides an estimate of right atrial pressure (RAP), but its correlation with invasive hemodynamics is only moderate and affected by ventilation, intrathoracic pressure, and body habitus [10], [11], [12]. The right internal jugular vein (RIJV) distensibility index has been validated against right heart catheterization. A distensibility index <66% during the Valsalva maneuver accurately predicted elevated RAP with excellent reproducibility, supporting RIJV ultrasound as a physiologically reliable tool for guiding therapy [13]. Combined strategies may provide additional value. In the CAVAL US-AHF pilot trial, daily management guided by both LUS and IVC reduced residual congestion at discharge and suggested a lower rate of HF readmissions at 90 days [14].

Two prior meta-analyses have examined this question. Li et al. (2022) found a significant readmission benefit with LUS-guided therapy but did not examine IVC assessment or compare modalities [15]. Al-Sagban et al. recently pooled nine RCTs with similar conclusions [16]. The present analysis, registered independently before either publication, extends this work by including combined LUS + IVC trials alongside LUS-only trials and applying a pre-specified modality-stratified subgroup analysis to directly compare the two ultrasound strategies. Given the growing adoption of ultrasound in clinical practice but inconsistent evidence regarding the differential impact of ultrasound modality on outcomes, we performed a systematic review and meta-analysis of randomized controlled trials. Our objective was to determine whether USG management using LUS, IVC, or combined LUS + IVC ultrasound reduces hospital readmissions and all-cause mortality in patients with HF as compared to standard care, and whether the benefit differs by modality.

## 2 Methodology

This systematic review and meta-analysis were conducted in accordance with the PRISMA 2020 guidelines and the AMSTAR 2 framework for assessing methodological quality. The protocol was developed before initiating the review process and submitted for prospective registration with PROSPERO prior to data extraction; a registration number had not been assigned at the time of manuscript submission.

### 2.1 Search strategy

A comprehensive literature search was performed across PubMed, Embase, Web of Science, and Google Scholar through January 2025. The search strategy was designed in collaboration with a medical librarian and included both controlled vocabulary (e.g., MeSH terms) and free-text keywords. Terms related to Point-of-Care Ultrasound (POCUS), HF, diuretic therapy, lung ultrasound, IVC ultrasound, and cardiac ultrasound were used. Boolean operators (“AND,” “OR”) and truncation symbols were applied to optimize sensitivity and specificity.

### 2.2 Study selection

Studies were eligible for inclusion if they were randomized controlled trials (RCTs) involving adult patients (≥18 years) diagnosed with acute or chronic HF. The intervention of interest was diuretic therapy guided by bedside ultrasound of the lung and/or IVC compared to standard care or diuretic therapy guided by clinical assessment alone. Eligible studies were required to report at least one of the following outcomes: all-cause mortality or HF-related hospitalization. Studies were excluded if they were observational, case reports, editorials, or reviews. Non-English publications, studies involving pediatric populations, and those using ultrasound solely for diagnostic purposes without guiding therapy were also excluded.

All retrieved citations were imported into Rayyan.ai for screening. Duplicate records were removed using automated tools. Two reviewers independently screened titles and abstracts, followed by a full-text review of potentially eligible studies. Discrepancies were resolved through discussion or consultation with a third reviewer. Data extraction was performed using a standardized Excel spreadsheet and included study characteristics (author, year, country, design), participant demographics, type of ultrasound modality used, and outcome measures. All extracted data were cross verified for accuracy and completeness.

### 2.3 Risk of bias measurement

Risk of bias was assessed independently by two reviewers using the Cochrane Risk of Bias 2.0 (ROB 2.0) tool. Domains evaluated included the randomization process, deviations from intended interventions, missing outcome data, measurement of the outcomes, and selection of reported results. Any disagreements were resolved through consensus or consultation with a third reviewer.

### 2.4 Data synthesis

Meta-analysis was conducted using Review Manager (RevMan) software. For dichotomous outcomes, pooled risk ratios (RR) with 95% confidence intervals (CI) were calculated using the DerSimonian–Laird random-effects model to account for clinical and methodological heterogeneity. Statistical heterogeneity was assessed using the I^2^ statistic, with values above 75% indicating substantial heterogeneity. Where appropriate, subgroup analyses were planned based on ultrasound modality (lung vs IVC or both) and care setting (inpatient vs. outpatient). A subgroup analysis by HF acuity (acute vs chronic HF) was considered; however, most trials enrolled patients with both acute decompensated and chronic HF or did not provide separately reported outcome data by HF acuity category, precluding a formal pooled comparison. This heterogeneity in clinical population is acknowledged as a limitation and a formal subgroup analysis by HF acuity was not feasible.

## 3 Results

### 3.1 Study selection

**Fig. 1:**
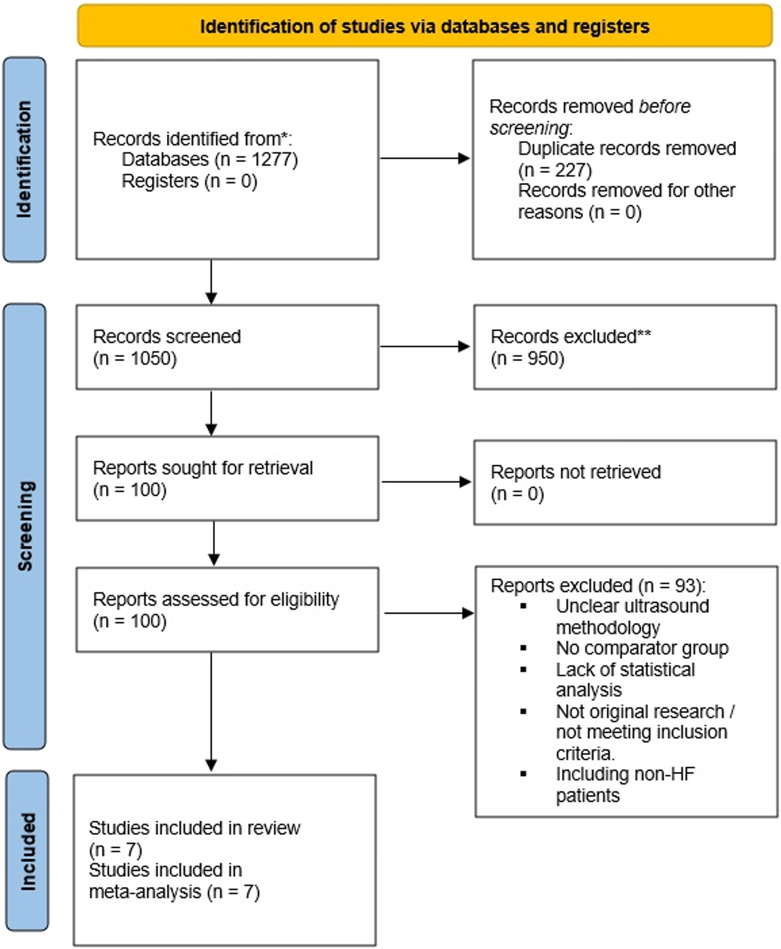
PRISMA 2020 flow diagram.

Following the initial search and removal of duplicates, 1050 records remained and were screened by title and abstract. Of these, 950 were excluded due to irrelevant populations (e.g., non–HF), use of only echocardiography without additional ultrasound modalities (such as lung or IVC ultrasound), absence of relevant outcomes (e.g., hospital readmission or mortality), or because they were not original research (e.g., reviews, editorials). Ultimately, seven randomized controlled trials met all inclusion criteria and were included in the qualitative synthesis and meta-analysis. The full selection process is illustrated in the PRISMA flow diagram (Fig. 1).

### 3.2 Study characteristics

The seven included RCTs, published between 2019 and 2024, enrolled adult patients with de novo or acute exacerbated chronic HF across six countries; mean ages ranged from 62.5 to 85 years, with 45.5–72% male participants.

All seven included studies were randomized controlled trials. The LUDT-ADHF study (Kashoob et al., 2025) was identified during full-text screening but excluded from both the quantitative meta-analysis and the qualitative synthesis, as it was non-randomized and open-label and therefore did not meet the pre-specified inclusion criteria. No risk-of-bias assessment was performed for this study.

Settings varied across inpatient (Burgos), hospital-at-home (Palacios García), and outpatient post-discharge models (Rivas-Lasarte, Araiza, Marini, Torres-Macho, Zisis). Ultrasound modalities comprised LUS alone in five trials and combined LUS + IVC in two (Palacios García and Burgos). All comparators reflected standard clinical care, with the caveat that Zisis et al. used a disease management programme (DMP) as comparator, and Torres-Macho et al. enrolled patients within a comprehensive multidisciplinary HF programme (UMIPIC), both representing enhanced rather than simple standard care.

Sample sizes in the ultrasound groups ranged from 30 to 127, with follow-up durations spanning 60 to 180 days.

### 3.3 Readmission outcomes

**Fig. 2:**
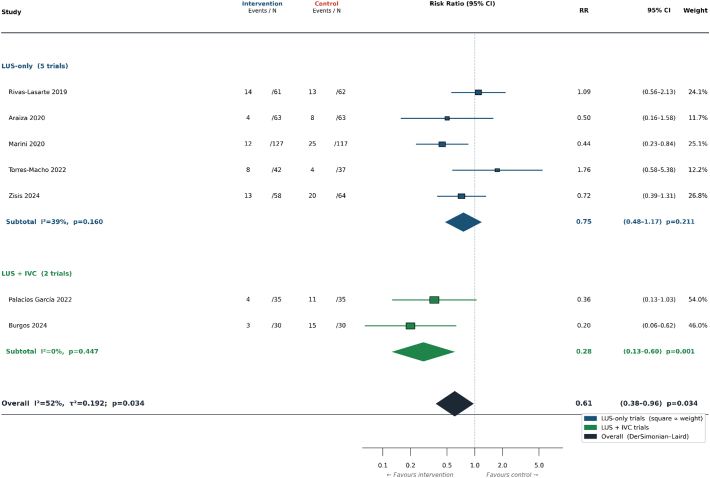
Forest plot of risk ratios for HF readmission ultrasound-guided vs standard care.

All seven RCTs reported HF hospitalization or readmission as a pre-specified endpoint; USG-based therapy was associated with numerically fewer events in five of the seven trials (Table 1, Fig. 2).

**Table 1:** Characteristics of the seven included randomized controlled trials.

Study (year, trial)	Country	Design and setting	US modality	Comparator	N (int / ctrl)	Mean age, y	Male, %	Follow-up, d
Rivas-Lasarte 2019 (LUS-HF)	Spain	Single-center, single-blind; ambulatory, post-discharge	LUS alone	Standard care	123 (61 / 62)	69	72	180
Araiza-Garaygordobil 2020 (CLUSTER-HF)	Mexico	Single-center, single-blind; outpatient, post-discharge	LUS alone	Usual care	126 (63 / 63)	62.5	69.5	180
Marini 2020	Italy and Armenia	Multicenter, open-label; outpatient, chronic HFrEF, no recent hospitalization	LUS (with PE)	Physical examination only	244 (127 / 117)	71.6	68	90
Torres-Macho 2022 (EPICC)	Spain	Multicenter, single-blind; outpatient, post-discharge (UMIPIC; stopped early)	LUS alone	Standard of care	79 (42 / 37)	81.2	45.5	180
Palacios Garcia 2022 (AHF-CU)	Spain	Single-center, open-label, parallel; hospital-at-home	LUS + IVC	Standard care	70 (35 / 35)	85.0	50	60
Burgos 2024 (CAVAL US-AHF)	Argentina	Single-center, single-blind, pilot; inpatient (AHF)	LUS + IVC	Standard care	60 (30 / 30)	76	68	90
Zisis 2024 (RISK-HF)	Australia	Multisite, open-label (PROBE), nurse-led; post-discharge, high-risk	LUS	Usual care (nurse-led DMP)	122 (58 / 64)	76	57	90

US, ultrasound; LUS, lung ultrasound; IVC, inferior vena cava; PE, physical examination; HFrEF, HF with reduced ejection fraction; AHF, acute heart failure; PROBE, prospective randomized open blinded end-point; DMP, disease management programme; UMIPIC, comprehensive multidisciplinary HF programme; int, intervention arm; ctrl, control arm. All values were taken from the source trials. N is the number analyzed with the intervention/control split; numbers randomized differ where there were losses before analysis: LUS-HF 124 randomized (63/61), CLUSTER-HF 128 randomized (65/63), AHF-CU 79 randomized (39/40). Reported mean age spans 62.5 (CLUSTER-HF) to 85.0 (AHF-CU); male sex spans 45.5% (EPICC) to 72% (LUS-HF); follow-up spans 60 (AHF-CU) to 180 days. *RISK-HF used a lungs US (LUICA) protocol but is classified under LUS alone in the subgroup analysis, consistent with how the trial reported its primary imaging-guided strategy.

The pooled risk ratio for HF readmission was 0.61 (95% CI: 0.38–0.96, *p* = 0.034), indicating a statistically significant 39% reduction in readmission with USG care, with moderate heterogeneity (I^2^ = 52%).

When trials were grouped by ultrasound modality, results differed substantially. The five LUS-only trials showed a non-significant trend toward fewer readmissions (RR 0.75, 95% CI 0.48–1.17, *p* = 0.211; I^2^ = 39%), whereas the two combined LUS + IVC trials together yielded a larger numerical reduction (RR 0.28, 95% CI 0.13–0.60, *p* = 0.001; I^2^ = 0%; 2 trials, *n* = 65 in the intervention arm). This difference should be treated cautiously: only two small trials contributed to the LUS + IVC estimate, the test for subgroup interaction did not reach statistical significance, and the finding is best regarded as hypothesis-generating pending confirmation in larger studies.

These results are illustrated in Fig. 2.

### 3.4 Mortality outcomes

**Fig. 3:**
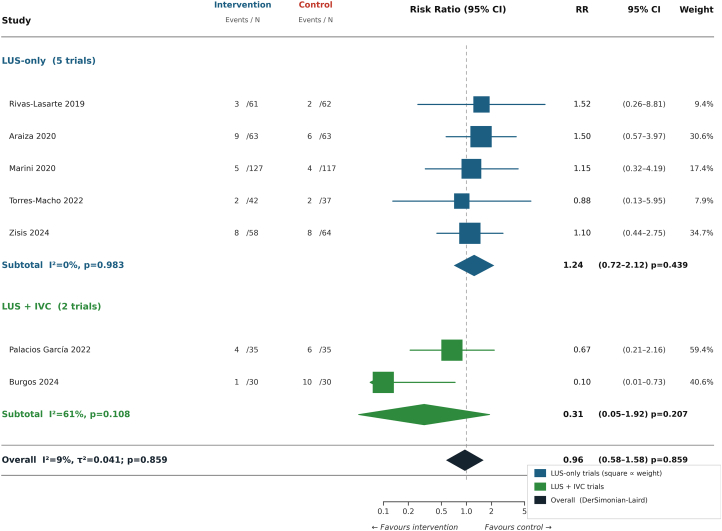
Forest plot of risk ratios for mortality using ultrasound guidance compared with standard care.

All seven RCTs reported all-cause mortality; USG-based therapy was associated with fewer deaths in three trials and numerically more in four, with no individual study reaching statistical significance (Fig. 3).

The pooled analysis showed no statistically significant reduction in all-cause mortality with USG therapy (RR 0.96, 95% CI: 0.58–1.58, *p* = 0.859), with low heterogeneity (I^2^ = 9%).

Subgroup analysis showed no significant difference in studies using LUS alone (RR 1.24, 95% CI: 0.72–2.12, *p* = 0.439; I^2^ = 0%) or combined LUS + IVC (RR 0.31, 95% CI: 0.05–1.92, *p* = 0.207; I^2^ = 61%). The difference between subgroups was not statistically significant.

Only one study, Burgos et al., demonstrated a statistically significant reduction in mortality at the individual study level. These results are illustrated in Fig. 3**.**

### 3.5 Sensitivity analysis

Three pre-specified sensitivity analyses were performed for the primary HF readmission outcome. First, excluding Zisis et al. (whose comparator was a structured disease management programme rather than standard care) yielded RR 0.58 (95% CI: 0.32–1.03, *p* = 0.064), which was attenuated to non-significance, reflecting the influence of the comparator definition. Second, excluding both Zisis et al. and Torres-Macho et al. (both enrolled within structured multidisciplinary outpatient programmes that may have attenuated the treatment effect) strengthened the result: RR 0.49 (95% CI: 0.28–0.86, *p* = 0.013; I^2^ = 51%). Third, excluding Burgos et al. (a pilot study in which clinical endpoints were explicitly exploratory) yielded RR 0.69 (95% CI: 0.45–1.05, *p* = 0.086), indicating that the primary result is sensitive to this single pilot trial. Sensitivity analyses for mortality did not alter the conclusion of no significant benefit across any model (Fig. 4a, Fig. 4b, Fig. 4c). A leave-one-out analysis confirmed that no single trial unduly dominated the overall estimate (Fig. 4d).

**Fig. 4a:**
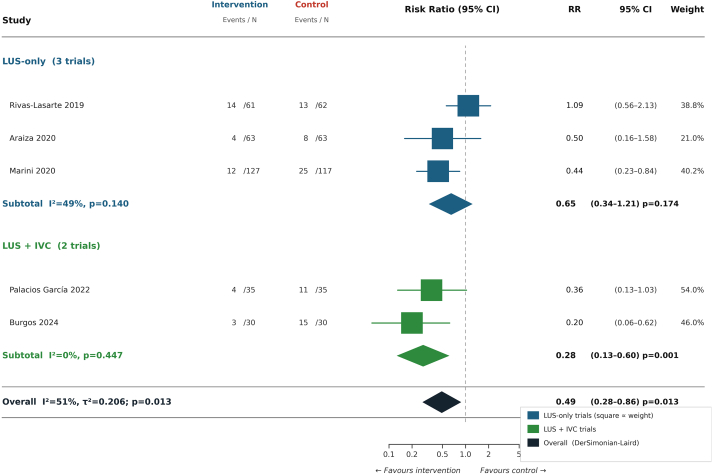
Sensitivity Analysis Excluding Torres-Macho 2022 and Zisis 2024. Forest Plot of Risk Ratios for HF Readmission.

**Fig. 4b:**
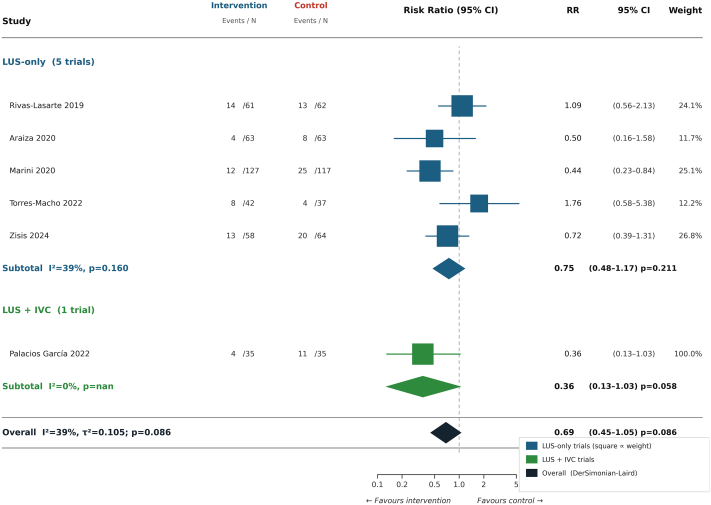
Sensitivity Analysis Excluding Burgos 2024 (Pilot Study). Forest Plot of Risk Ratios for HF Readmission.

**Fig. 4c:**
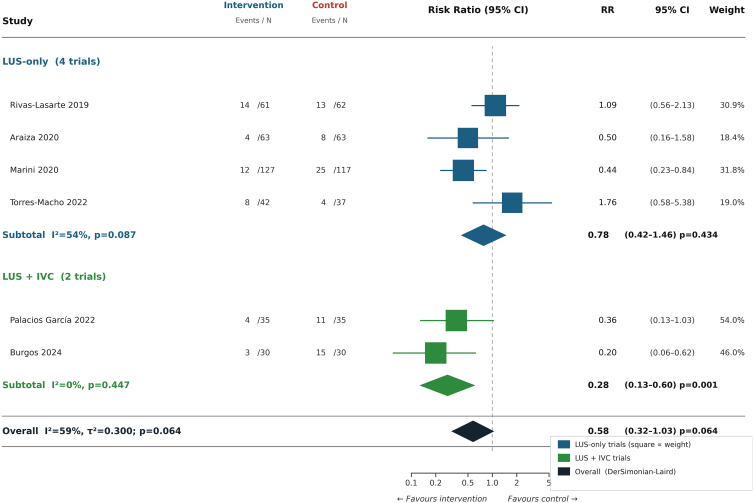
Sensitivity Analysis Excluding Zisis 2024 (RISK-HF; structured DMP comparator). Forest Plot of Risk Ratios for HF Readmission.

**Fig. 4d:**
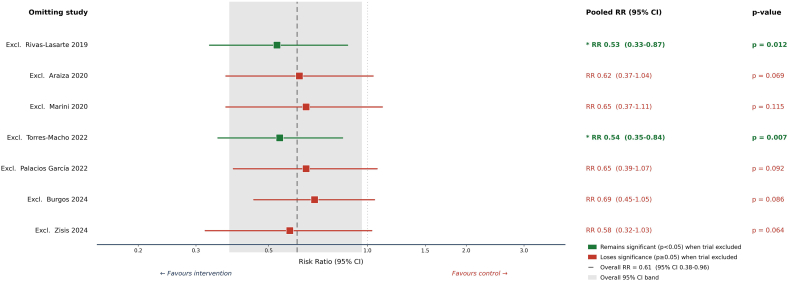
Leave-One-Out Sensitivity Analysis HF Hospitalization/Readmission. Pooled RR (95% CI) when each trial is excluded in turn; green markers = result remains significant, red = significance lost. (For interpretation of the references to colour in this figure legend, the reader is referred to the web version of this article.)

### 3.6 Secondary findings

Secondary outcomes related to clinical decision-making, medication changes, or healthcare utilization were not consistently reported across the included studies. While several trials mentioned the use of lung or IVC ultrasound to assess volume status, there was no structured data on changes in diuretic dosing, hospital length of stay, medication adjustments, or escalation of care.

Most studies used handheld or ultraportable devices with 8-zone LUS protocols, with or without IVC assessment; no study formally evaluated feasibility, workflow impact, or cost-effectiveness.

Due to these limitations in reporting, secondary outcomes could not be pooled quantitatively. Nevertheless, the available information suggests that POCUS may play a role in tailoring volume management, although further research is needed to clarify its broader clinical and economic impact.

### 3.7 Risk of bias and certainty of evidence

A summary of all pooled results, subgroup analyses, and sensitivity analyses is presented in Table 2. All seven included randomized controlled trials were assessed using the Cochrane Risk of Bias 2.0 (ROB 2.0) tool. These included Rivas-Lasarte et al. 2019 (LUS-HF) [8], Araiza-Garaygordobil et al. 2020 (CLUSTER-HF) [17], Marini et al. 2020 [18], Torres-Macho et al. 2022 (EPICC) [19], Palacios García et al. 2022 (AHF-CU) [20], Burgos et al. 2024 (CAVAL US-AHF) [14], and Zisis et al. 2024 (RISK-HF) [21].

**Table 2:** Summary of pooled, subgroup, and sensitivity analyses.

Analysis	Trials, n	Risk ratio (95% CI)	p	I^**2**^, %
HF hospitalization / readmission				
Overall pooled estimate	7	0.61 (0.38 to 0.96)	0.034	52
Subgroup: LUS alone	5	0.75 (0.48 to 1.17)	0.211	39
Subgroup: LUS + IVC	2	0.28 (0.13 to 0.60)	0.001	0
Sensitivity: excluding Zisis 2024	6	0.58 (0.32 to 1.03)	0.064	59
Sensitivity: excluding Zisis 2024 and Torres-Macho 2022	5	0.49 (0.28 to 0.86)	0.013	51
Sensitivity: excluding Burgos 2024 (pilot)	6	0.69 (0.45 to 1.05)	0.086	39

All-cause mortality				
Overall pooled estimate	7	0.96 (0.58 to 1.58)	0.859	9
Subgroup: LUS alone	5	1.24 (0.72 to 2.12)	0.439	0
Subgroup: LUS + IVC	2	0.31 (0.05 to 1.92)	0.207	61
Sensitivity analyses (all models)	n/a	No significant benefit across any model	n/a	n/a

RR, risk ratio; CI, confidence interval; LUS, lung ultrasound; IVC, inferior vena cava; HF, heart failure; n/a, not applicable. Random-effects models. All values are as reported in the manuscript Results. GRADE certainty of evidence was rated low for both readmission and mortality. Subgroup interaction tests were not statistically significant for either outcome.

All seven trials were rated as having some concern for performance bias due to the inherent inability to blind participants and treating clinicians to ultrasound findings a common and expected limitation in POCUS intervention trials. All seven trials adequately performed random sequence generation, allocation concealment, and blinding of outcome adjudicators. Other domains were generally assessed as low or low-to-moderate risk.

Using GRADE, the certainty of evidence was rated as low for both mortality and readmission. Downgrades were applied for risk of bias (performance bias due to inherent lack of blinding in ultrasound intervention trials), inconsistency for readmission (moderate heterogeneity, I^2^ = 52%), and imprecision for mortality (wide confidence interval and limited number of events). Risk-of-bias assessments are presented in Fig. 5a; and the ROB domain summary is presented in Fig. 5b.

**Fig. 5a:**
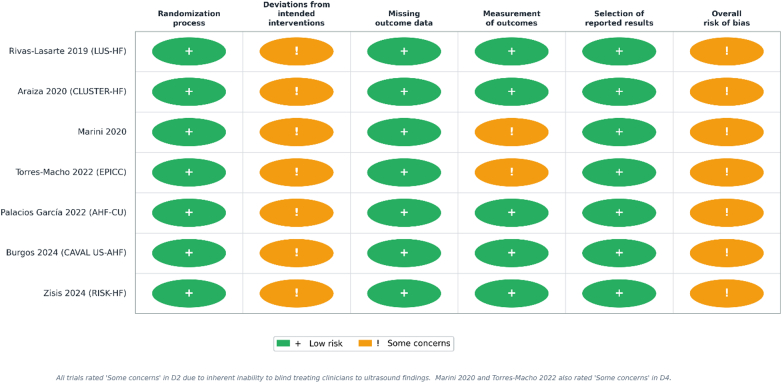
Risk of Bias Assessment All 7 RCTs (Cochrane ROB 2.0 Tool). Outcomes: HF Hospitalization and All-Cause Mortality.

**Fig. 5b:**
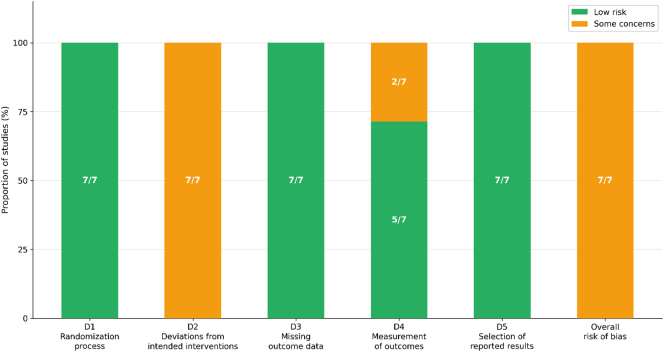
Risk of Bias Summary Distribution Across ROB 2.0 Domains (7 RCTs).

## 4 Discussion: ultrasound-guided management in heart failure – focus on readmission and mortality outcomes

This systematic review and meta-analysis of seven RCTs involving 824 patients demonstrates that USG management significantly reduces HF hospital readmissions (RR 0.61; 95% CI 0.38–0.96; *p* = 0.034; I^2^ = 52%) but does not significantly impact all-cause mortality (RR 0.96; 95% CI 0.58–1.58; *p* = 0.859; I^2^ = 9%). These findings are broadly consistent with two concurrently published meta-analyses: Chotalia et al. (2026) demonstrated similar readmission benefits in patients with chronic HF [22], and Al-Sagban et al. (2026) synthesized nine RCTs across both acute and chronic HF populations [16]. The present analysis extends this evidence base by incorporating a modality-stratified subgroup analysis and a pre-specified sensitivity analysis framework. Of note, Al-Sagban et al. (2026) [16] included nine RCTs compared to our seven. The two additional trials in their analysis employed different follow-up endpoints or inclusion frameworks that did not satisfy our pre-specified criteria requiring clinical outcomes of all-cause mortality or HF-related readmission as primary endpoints and the use of ultrasound to guide therapeutic decisions rather than for diagnostic purposes only.

The 39% reduction in HF readmissions (RR 0.61; *p* = 0.034) underscores the clinical utility of USG management in addressing a major driver of HF morbidity and healthcare costs [2], [23]. Ultrasound enables precise, real-time assessment of volume status and pulmonary congestion, facilitating tailored diuretic optimization [24], [25]. LUS detects pulmonary edema through B-lines, while IVC ultrasound assesses central venous pressure and systemic fluid status, providing complementary insights into HF pathophysiology [26], [27], [28]. A numerically larger reduction was observed with combined LUS + IVC (RR 0.28; 95% CI 0.13–0.60; *p* = 0.001; 2 trials, *n* = 65 intervention arm). However, the test for subgroup interaction was not statistically significant, and this finding should be interpreted as hypothesis-generating rather than confirmatory, pending evaluation in adequately powered dedicated trials [17], [29]. For example, a patient with B-lines and a distended, non-collapsible IVC may require aggressive diuresis, whereas isolated B-lines might prompt conservative management [13], [30].

In contrast, LUS alone showed a non-significant trend toward reduced readmissions (RR 0.75; *p* = 0.211), with wider confidence intervals (0.48–1.17). This indicates that while LUS is valuable for detecting pulmonary vascular congestion, it may not fully assess right-sided filling pressures, which IVC ultrasound addresses through parameters like diameter and collapsibility [7], [8], [31]. These findings suggest that combined LUS + IVC assessment may offer advantages over either modality alone, though this hypothesis requires prospective evaluation in trials designed to directly compare the two strategies [10], [11]. The CAVA-ADHF-DZHK10 trial (*n* = 388), the largest IVC-guided RCT to date, found no improvement in NT-proBNP reduction when IVC ultrasound was used without concurrent lung assessment [32], reinforcing the potential value of the combined approach.

The absence of a significant effect on all-cause mortality (RR 0.96; *p* = 0.859; I^2^ = 9%) may be attributed to several factors. First, the pooled sample of 824 patients limits statistical power to detect differences in mortality, a less frequent outcome than readmissions [25], [33]. Second, HF mortality is influenced by factors beyond volume status, including comorbidities, adherence to guideline-directed medical therapy, and neurohormonal activation, which USG care may not directly address [25], [34]. The low heterogeneity (I^2^ = 9%) indicates this null result is consistent across all seven trials rather than a product of conflicting data. Future analyses incorporating individual patient data, stratified by ejection fraction phenotype, may be better positioned to detect mortality effects in specific subgroups [25], [35].

The clinical implications of these findings are significant. The robust reduction in readmissions, particularly with combined lung and IVC ultrasound, supports its integration into routine HF care, especially in settings with high rehospitalization rates [25], [28]. Ultrasound is non-invasive, cost-effective, and portable, making it feasible for use in outpatient clinics, emergency departments, and hospital-at-home settings. The 2023 EACVI consensus statement on lung ultrasound in HF provides a structured framework for implementation, including standardized zone protocols, B-line thresholds, and documentation requirements that can be incorporated into existing workflows [28]. Training clinicians in both lung and IVC ultrasound techniques could enhance adoption and improve outcomes in high-risk populations [25], [28].

Several research priorities emerge from this analysis. First, adequately powered multicenter RCTs are needed, particularly given the projected growth in HF prevalence [36], to determine whether the readmission benefit extends to HF patients with preserved ejection fraction, who were underrepresented across the included trials. Second, studies should define the optimal ultrasound assessment frequency, the minimum clinician training requirement, and the performance characteristics of automated B-line quantification tools that may reduce inter-operator variability [27]. Third, formal cost-effectiveness analyses are required before health systems can justify widespread protocol implementation, particularly given that repeated HF hospitalizations are independently associated with worsening long-term prognosis [37].

**Fig. 6:**
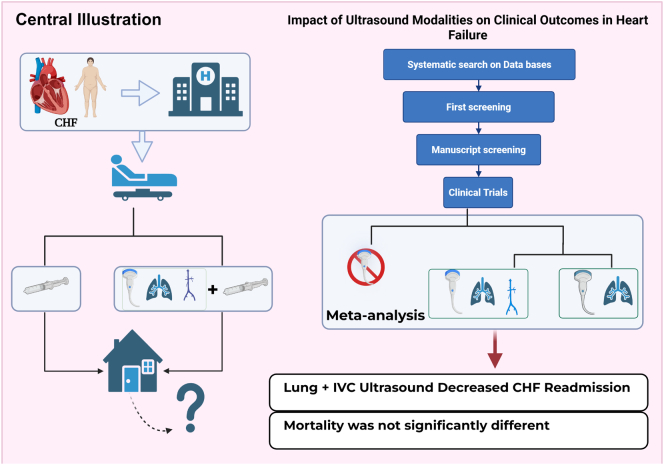
Central illustration [39].

Several limitations affect how confidently these results can be generalised. The combined enrolment of 824 patients across seven trials is modest; for readmission this was sufficient to detect a statistically significant effect, but for mortality the analysis was substantially underpowered and a null result should not be interpreted as evidence of no effect. The primary readmission finding is fragile in one particular respect: removing the Burgos pilot trial the only inpatient study, with explicitly exploratory clinical endpoints shifts the pooled result to non-significance, which means the current evidence base rests partly on a single small study not designed to provide definitive outcome data. Two additional trial-level concerns deserve mention: Marini et al. was stopped early at an interim analysis showing overwhelming benefit, a design feature that tends to inflate treatment effect estimates; and Zisis et al. used a structured nurse-led disease management programme as the comparator, raising questions about whether patients in the control arm received a level of care substantially better than typical standard practice, which would attenuate the apparent treatment benefit. Beyond individual trial issues, the included studies varied widely in scan protocols, device types, operator training standards, follow-up duration, and clinical setting (inpatient, hospital-at-home, and post-discharge outpatient), all of which limit the homogeneity of the pooled estimate. The deliberate exclusion of echocardiography-guided studies, while necessary to isolate the effect of LUS and IVC assessment specifically, means these findings say nothing about whether broader imaging-guided strategies would be more or less effective [38]. Finally, most included trials enrolled patients with both acute decompensated and chronic HF without reporting outcomes separately by HF acuity; a pre-specified subgroup analysis was planned but was not feasible given this reporting structure, leaving uncertainty about whether the benefit is concentrated in one clinical phenotype (Fig. 6).

## 5 Conclusion

This meta-analysis provides evidence that integrating ultrasound assessment into HF management significantly reduces hospital readmissions across seven RCTs (*n* = 824). A hypothesis-generating signal of greater benefit with combined lung and IVC ultrasound was observed, suggesting that simultaneous assessment of pulmonary and systemic venous congestion may enable more precise decongestion; however, this finding is based on only two small trials (*n* = 65 intervention arm) and requires confirmation in adequately powered prospective trials before it can inform practice recommendations. The absence of a mortality benefit is consistent across all seven trials and reflects the multifactorial nature of HF survival beyond volume management. Point-of-care ultrasound is already available in most HF care settings, and the 2023 EACVI consensus provides a structured implementation framework. Future trials should enrol HFpEF patients, define standardized protocols, and include cost-effectiveness evaluation.

## CRediT authorship contribution statement

**Amir Behzad Bagheri:** Writing – review & editing, Writing – original draft, Validation, Methodology, Conceptualization. **Muhammad Saleem:** Writing – original draft, Formal analysis, Data curation. **Sina Bakhshaei:** Writing – original draft, Data curation. **Raymond Andrew Dieter:** Writing – original draft, Data curation. **Ali Ajam:** Data curation. **Yasaman Navari:** Writing – review & editing, Writing – original draft. **Benay Ozbay:** Writing – original draft, Data curation. **Soham Nadkarni:** Writing – original draft. **Parth Patel:** Writing – original draft, Data curation. **Mobin Kheirkhahan:** Data curation. **John Jude Pacella:** Writing – review & editing, Conceptualization. **Babak Razani:** Validation, Supervision.

## Ethical approval and consent to participate

This study is a systematic review and meta-analysis of previously published data and does not involve direct human or animal participation. Therefore, institutional review board approval and informed consent were not required.

## Ethical compliance

All studies included in this analysis were conducted in accordance with the ethical standards of their respective institutions.

## Declaration of competing interest

The authors declare that they have no known competing financial interests or personal relationships that could have appeared to influence the work reported in this paper.

## References

[1] Bozkurt B., Ahmad T., Alexander K.M., Bhatt A.S., Bluemke D.A., Celermajer D.S. (2023). Heart failure epidemiology and outcomes statistics: a report of the Heart Failure Society of America. J. Card. Fail..

[2] Jencks S.F., Williams M.V., Coleman E.A. (2009). Rehospitalizations among patients in the Medicare fee-for-service program. N. Engl. J. Med..

[3] Stevenson L.W., Perloff J.K. (1989). The limited reliability of physical signs for estimating hemodynamics in chronic heart failure. JAMA.

[4] McDonagh T.A., Metra M., Adamo M., Gardner R.S., Baumbach A., Böhm M. (2023). Focused update of the 2021 ESC guidelines for the diagnosis and treatment of acute and chronic heart failure. Eur. J. Heart Fail. 2024.

[5] Pellicori P., Platz E., Dauw J., Ter Maaten J.M., Martens P., Pivetta E. (2021). Ultrasound imaging of congestion in heart failure: examinations beyond the heart. Eur. J. Heart Fail..

[6] Martindale J.L., Wakai A., Collins S.P., Levy P.D., Diercks D., Hiestand B.C. (2016). Diagnosing acute heart failure in the emergency department: a systematic review and meta-analysis. Acad. Emerg. Med..

[7] Volpicelli G., Elbarbary M., Blaivas M., Lichtenstein D.A., Mathis G., Kirkpatrick A.W. (2012). International evidence-based recommendations for point-of-care lung ultrasound. Intensive Care Med..

[8] Rivas-Lasarte M., Álvarez-García J., Fernández-Martínez J., Maestro A., López-López L., Solé-González E. (2019). Lung ultrasound-guided treatment in ambulatory patients with heart failure: a randomized controlled clinical trial (LUS-HF study). Eur. J. Heart Fail..

[9] Pang P.S., Russell F.M., Ehrman R., Coutts B., Dusak B., Hiestand B. (2021). Lung ultrasound-guided emergency department management of acute heart failure (BLUSHED-AHF): a randomized controlled pilot trial. JACC Heart Fail..

[10] Di Nicolò P., Tavazzi G., Nannoni L., Corradi F. (2023). Inferior vena cava ultrasonography for volume status evaluation: an intriguing promise never fulfilled. J. Clin. Med..

[11] Vieillard-Baron A., Evrard B., Repressé X., Maizel J., Jacob C., Goudelin M. (2018). Limited value of end-expiratory inferior vena cava diameter to predict fluid responsiveness impact of intra-abdominal pressure. Intensive Care Med..

[12] Brennan J.M., Blair J.E., Goonewardena S., Ronan A., Shah D., Vasaiwala S. (2007). A comparison by medicine residents of physical examination versus hand-carried ultrasound for estimation of right atrial pressure. Am. J. Cardiol..

[13] Simon M.A., Kliner D.E., Girod J.P., Moguillansky D., Villanueva F.S., Pacella J.J. (2010). Detection of elevated right atrial pressure using a simple bedside ultrasound measure. Am. Heart J..

[14] Burgos L.M., Baro Vila R.C., Ballari F.N., Charask A., Vico M., Alvarez Bello F. (2024). Inferior vena cava and lung ultrasound-guided therapy in acute heart failure: a randomized pilot study (CAVAL US-AHF study). Am. Heart J..

[15] Li Y., Ai H., Ma N., Li P., Ren J. (2022). Lung ultrasound-guided treatment for heart failure: an updated meta-analysis and trial sequential analysis. Front. Cardiovasc. Med..

[16] Al-Sagban A., Algodi M., Saab O., Al-Obaidi H., Graham H., Abuelazm M.T. (2026). Lung ultrasound-guided decongestion in heart failure patients: a systematic review and meta-analysis of randomized controlled trials. J. Crit. Care.

[17] Araiza-Garaygordobil D., Gopar-Nieto R., Martinez-Amezcua P., Cabello-López A., Alanis-Estrada G., Luna-Herbert A. (2020). A randomized controlled trial of lung ultrasound-guided therapy in heart failure (CLUSTER-HF study). Am. Heart J..

[18] Marini C., Fragasso G., Italia L., Sisakian H., Tufaro V., Ingallina G. (2020). Lung ultrasound-guided therapy reduces acute decompensation events in chronic heart failure. Heart.

[19] Torres-Macho J., Cerqueiro-González J.M., Arévalo-Lorido J.C., Llácer-Iborra P., Cepeda-Rodrigo J.M., Cubo-Romano P. (2022). The effects of a therapeutic strategy guided by lung ultrasound on 6-month outcomes in patients with heart failure: results from the EPICC randomized controlled trial. J. Clin. Med..

[20] Palacios García L., Enguita Germán M., Ruiz Sada P., Echeverría Echeverría A., González Gómez M., Rubio Obanos M.T. (2022). Impact of clinical ultrasound in patients with heart failure treated in home hospitalization. Med. Clin. (Barc.).

[21] Zisis G., Carrington M.J., Yang Y., Huynh Q., Lay M., Whitmore K. (2024). Use of imaging-guided decongestion for reducing heart failure readmission and death in high-risk patients: a multi-site randomized trial of a nurse-led strategy at the point of care. J. Card. Fail..

[22] Chotalia R., Mohee K., Ambrogetti R., Chotalia M., Rahman L.R., Mohiaddin H. (2026). Lung ultrasound guided management in chronic heart failure: an updated systematic review and meta-analysis of randomized controlled trials. Eur. Heart J. Imag. Methods Pract..

[23] Gheorghiade M., Vaduganathan M., Fonarow G.C., Bhatt D.L., Butler J., Filippatos G. (2013). Rehospitalization for heart failure: problems and perspectives. J. Am. Coll. Cardiol..

[24] Platz E., Campbell R.T., Claggett B., Lewis E.F., Groarke J.D., Docherty K.F. (2019). Lung ultrasound in acute heart failure: prevalence of pulmonary congestion and short- and long-term outcomes. JACC Heart Fail..

[25] Heidenreich P.A., Bozkurt B., Aguilar D., Allen L.A., Byun J.J., Colvin M.M. (2022). 2022 AHA/ACC/HFSA guideline for the management of heart failure. Circulation.

[26] Lala A., SE McNulty, Mentz R.J., Dunlay S.M., Vader J.M., Fort Arnold J. (2015). Relief and recurrence of congestion during and after hospitalization for acute heart failure: insights from DOSE-AHF and CARESS-HF. Circ. Heart Fail..

[27] Platz E., Jhund P.S., Girerd N., Pivetta E., McMurray J.J.V., Peacock W.F. (2019). Expert consensus document: reporting checklist for quantification of pulmonary congestion by lung ultrasound in heart failure. Eur. J. Heart Fail..

[28] Gargani L., Girerd N., Platz E., Pellicori P., Stankovic I., Palazzuoli A. (2023). Lung ultrasound in acute and chronic heart failure: a clinical consensus statement of the European Association of Cardiovascular Imaging (EACVI). Eur. Heart J. Cardiovasc. Imaging.

[29] Pellicori P., Shah P., Cuthbert J., Kazmi S., Clark A.L., JGF Cleland (2019). Prevalence, pattern and clinical relevance of ultrasound indices of congestion in outpatients with heart failure. Eur. J. Heart Fail..

[30] Coiro S., Rossignol P., Ambrosio G., Carluccio E., Alunni G., Murrone A. (2015). Prognostic value of residual pulmonary congestion at discharge assessed by lung ultrasound imaging in heart failure. Eur. J. Heart Fail..

[31] Blecker S., Paul M., Taksler G., Ogedegbe G., Katz S. (2013). Heart failure-associated hospitalizations in the United States. J. Am. Coll. Cardiol..

[32] Jobs A., Rausch T.K., König I.R., Vonthein R., Devendra A., Schäfer J. (2025). Inferior vena cava ultrasound to guide decongestion in acute decompensated heart failure: a randomized controlled trial. JACC Heart Fail..

[33] Ambrosy A.P., Fonarow G.C., Butler J., Chioncel O., Greene S.J., Vaduganathan M. (2014). The global health and economic burden of hospitalizations for heart failure: lessons learned from hospitalized heart failure registries. J. Am. Coll. Cardiol..

[34] Solomon S.D., Dobson J., Pocock S., Skali H., McMurray J.J.V., Granger C.B. (2007). Influence of nonfatal hospitalization for heart failure on subsequent mortality in patients with chronic heart failure. Circulation.

[35] Pitt B., Pfeffer M.A., Assmann S.F., Boineau R., Anand I.S., Claggett B. (2014). Spironolactone for heart failure with preserved ejection fraction. N. Engl. J. Med..

[36] Heidenreich P.A., Albert N.M., Allen L.A., Bluemke D.A., Butler J., Fonarow G.C. (2013). Forecasting the impact of heart failure in the United States: a policy statement from the American Heart Association. Circ. Heart Fail..

[37] Setoguchi S., Stevenson L.W., Schneeweiss S. (2007). Repeated hospitalizations predict mortality in the community population with heart failure. Am. Heart J..

[38] Neskovic A.N., Edvardsen T., Galderisi M., Garbi M., Gullace G., Hagendorff A. (2014). Focus cardiac ultrasound: the European Association of Cardiovascular Imaging viewpoint. Eur. Heart J. Cardiovasc. Imaging.

[39] Bagheri AB. (2026). http://BioRender.com.

